# Aspergillus oesophagitis in a patient with solid tumors: a case report

**DOI:** 10.1099/acmi.0.000842.v4

**Published:** 2024-09-13

**Authors:** Vanessa Abi Rached, Karine Azar, Sarah Gerges, Souheil Hallit, Elie Akoury, Georges Chahine, Rabih Hallit, Bassem Akiki, Rita Rizk

**Affiliations:** 1 School of Medicine and Medical Sciences, Holy Spirit University of Kaslik, P. O. Box 446, Jounieh, Lebanon; 2 Research Department, Psychiatric Hospital of the Cross, Jal Eddib, Lebanon; 3 Applied Science Research Center, Applied Science Private University, Amman, Jordan; 4 Department of Oncology, Notre Dame des Secours University Hospital Center, Street 93, Byblos, Postal Code 3, Lebanon; 5 Department of Infectious Disease, Notre Dame des Secours University Hospital Center, Street 93, Byblos, Postal Code 3, Lebanon; 6 Department of Infectious Disease, Bellevue Medical Center, Mansourieh, Lebanon; 7 Department of Gastroenterology, Notre Dame des Secours University Hospital Center, Street 93, Byblos, Postal Code 3, Lebanon

**Keywords:** *Aspergillus*, Aspergillosis, case report, gastrointestinal neoplasms, Lebanon

## Abstract

Oesophageal aspergillosis is a rare occurrence primarily documented in hematologic malignancies, and only rarely occurring among patients with solid tumours. In this case report, we present the unique case of an 81-year-old Lebanese man who had a remarkable medical history, including four solid tumours. The patient sought medical attention due to dysphagia and weight loss, prompting a gastroscopic examination that revealed a necrotic abscess at the oesophagogastric junction. Initial treatment with fluconazole and esomeprazole was administered, but the recurrence of similar symptoms led to a repeat gastroscopy, unveiling a diagnosis of *Aspergillus* oesophagitis. Intravenous voriconazole was promptly initiated; however, the patient developed a significant pericardial effusion and expired, with *Aspergillus* species identified in the pericardial fluid prior to patient expiring. This exceptional case emphasizes the importance of considering oesophageal aspergillosis in cancer patients who present with refractory symptoms such as epigastric pain, dysphagia, nausea, and vomiting, despite symptomatic treatment. Our findings underscore the need for increased awareness and the inclusion of gastrointestinal endoscopy as part of the diagnostic approach for this rare but potentially life-threatening condition.

## Data Summary

All data supporting the conclusions of this study are available upon reasonable request from the corresponding author. However, the authors are not allowed to publicly share some data due to restriction from the ethical committee, as per their institution’s policy; since clinical data involving human subjects adheres to institutional ethical guidelines and consent restrictions that limit public availability.

## Introduction

*Aspergillus*, a saprotrophic fungus primarily encountered in soil, has developed adaptive mechanisms to thrive in various hostile environments, including the human body with its immunological defenses [1]. Aspergillosis manifests in diverse forms, ranging from allergic to chronic and invasive, predominantly affecting the lower respiratory tract but also compromising the sino-nasal and upper respiratory regions [2]. Extrapulmonary aspergillosis, observed in 25–60% of disseminated aspergillosis cases, rarely involves the gastrointestinal region [2,3]. However, when the upper digestive tract is affected, it is attributed to the inhalation or ingestion of *Aspergillus* spores, leading to isolated aspergillosis [2]. Nonetheless, gastrointestinal aspergillosis is seldom diagnosed prior to autopsy and is believed to arise from fungal dissemination from the primary infection site into the bloodstream [1].

Consequently, invasive aspergillosis poses a significant challenge as a fatal infectious complication, particularly among immunocompromised hosts and especially those diagnosed with malignancies [1]. Hematologic malignancies, in particular, raise concerns due to the frequent association with high mortality rates in the context of aspergillosis [1]. Notably, the incidence of IA in leukaemia patients ranges from 15–20%, primarily attributed to mucosal damage, prolonged neutropenia, and the administration of immunosuppressive drugs [1]. The exceedingly poor prognosis in disseminated aspergillosis stems from the simultaneous occurrence of multiple infection sites [4].

While oesophageal involvement of aspergillosis has been documented in hematologic malignancies such as myeloblastic and lymphoblastic leukemias or following hematopoietic cell transplants [5,8], it only rarely occurs among patients with solid tumours. While exact incidence rates vary, it can cause severe symptoms like difficulty swallowing, chest pain, and bleeding, and is considered a significant cause of morbidity and mortality in this vulnerable population [9]. The infection often presents with debilitating symptoms, underscoring the need for early diagnosis and aggressive management [9]. In this report, we present a unique case of *Aspergillus* oesophagitis in a cancer patient diagnosed with four solid tumours.

## Case presentation

**Fig. 1.**
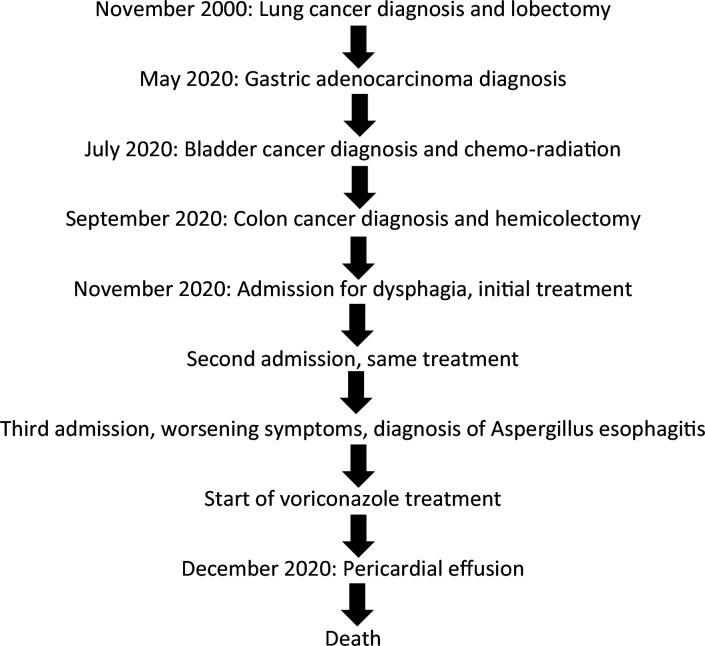
Overview of key milestones, treatments, and clinical outcomes.

A timeline summarizing key clinical milestones, diagnostic procedures, and therapeutic interventions is presented in Fig. 1.

### Patient information

We present the case of an 81-year-old Lebanese man presenting with severe progressive dysphagia and weight loss, who had a history of four solid tumours.

### Clinical findings and physical examination

Upon examination, the patient had no fever. His blood pressure was 100/80 mmHg, pulse was 110 beats min^−1^, and respiratory rate was 13 breaths min^−1^. The inspection of the oral cavity and throat did not reveal signs of oral thrush or other visible abnormalities. However, the neck was tender to palpation, and multiple enlarged lymph node were palpated. There were no abnormal heart sounds and no crackles. The abdomen was tender to deep and superficial palpation, with a palpable gastric mass and no evidence of guarding.

### Past medical history

The patient had a past medical history of an early-stage squamous cell carcinoma of the lung, which was managed through a lobectomy in November 2000. Subsequently, he was lost to follow-up until 2020 when three successive cancers were discovered. In May 2020, he presented with melena and anaemia, and a gastroscopy identified an ulcer in the gastric cardia. Biopsies confirmed the presence of an advanced-stage, intestinal-type adenocarcinoma. In July 2020, investigations revealed a high-grade urothelial carcinoma of the bladder with prostatic infiltration, which was managed using chemo-radiation. In September 2020, an abdominal and pelvic Magnetic Resonance Imaging revealed wall thickening in the sigmoid colon. A subsequent colonoscopy confirmed a locally-advanced, moderately differentiated adenocarcinoma (pT4aN1a), which might be a progression of the intestinal-type adenocarcinoma. The patient underwent a hemicolectomy for management. To our knowledge, the patient had no other medical comorbidities and no remarkable family history.

### Timeline of current episode (*Aspergillus* oesophagitis)

On 2 November 2020: the patient was admitted to the hospital due to severe progressive dysphagia and weight loss. Considering the patient’s history of radiation, a presumed diagnosis of radiation oesophagitis was considered. Initial treatment with esomeprazole 40 mg IV daily and sucralfate 1000 mg PO daily was initiated for 10 days to manage symptoms associated with presumed radiation oesophagitis. Fluconazole 400 mg IV daily was also administered empirically to cover for potential fungal oesophagitis given the patient’s risk factors. A subsequent gastroscopy uncovered a necrotic circumferential ulcer at the oesophagogastric junction, extending 5 cm from the Z-line (or gastro-oesophageal junction [GEJ] that represents the transition between the squamous epithelium of the oesophagus and the columnar epithelium of the stomach).

Two weeks later, on 18 November 2020: the patient returned with similar complaints, but he responded well to the same treatment regimen and was discharged.

Four days later, on 22 November 2020: the third presentation with comparable symptoms led to a repeat gastroscopy revealing a similar appearance, but with the necrotic ulcer now extending 7 cm from the Z line.

### Diagnostic assessment and laboratory findings

**Fig. 2.**
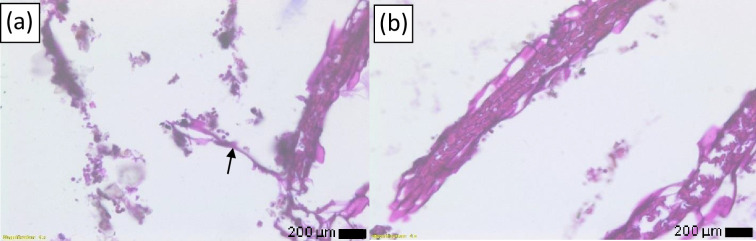
(a, b) Pathology analysis with PAS staining revealing fungal hyphae (arrow) consistent with *Aspergillus* species.

Blood tests were indicative of an infection: white blood cells count=18 300 cells mm^−^³, elevated neutrophils with a left shift, and C-reactive Protein (CRP)=130 mg l^−1^. In addition, multiple biopsies were taken during the second gastroscopy (on 22 November 2020). The pathology analysis, using periodic acid-Schiff (PAS) staining, confirmed the presence of *Aspergillus* species, characterized by septate hyphae exhibiting acute-angle, dichotomous branching (Fig. 2). A fungal culture was conducted on the oesophageal biopsy, on Sabouraud dextrose agar (BD, USA) at 37 °C for 7 days, aiding in the diagnosis of *Aspergillus* oesophagitis. While dichotomous branching can also be observed in other fungal genera such as Fusarium, Penicillium, and Scedosporium, the diagnosis of *Aspergillus* oesophagitis in this case was based on a combination of clinical presentation, characteristic pathological findings, and positive fungal culture.

### Diagnosis

The diagnosis of *Aspergillus* oesophagitis was established based on the presence of *Aspergillus* species confirmed through pathology analysis and fungal culture of the biopsy.

### Therapeutic interventions

Immediate initiation of intravenous voriconazole at a dose of 6 mg kg^−1^ every 12 h for two doses, followed by a maintenance dose of 4 mg kg^−1^ every 12 h for a prospective duration of 4 weeks, was instituted.

### Follow-up and outcome of interventions

Few days after starting voriconazole, the patient developed a significant pericardial effusion, most probably due to an oesophageal perforation. Pericardiocentesis was performed, and fluid analysis did not indicate malignancy but revealed fungal hyphae with dichotomous branching, consistent with *Aspergillus* species in the pericardial fluid. Unfortunately, the patient succumbed to a cardiac arrest a few days later.

## Discussion and review of the literature

This case presents a rare and unusual occurrence of *Aspergillus* oesophagitis in a patient with a history of multiple solid tumours, highlighting the need to expand the understanding of fungal infections in immunocompromised individuals. Within the literature, gastrointestinal involvement of *Aspergillus* has indeed been a rare occurrence, with only a handful of cases reported in leukaemia patients rather than cancer patients with solid tumours [7]. The susceptibility of these patients to fungal infections has been largely attributed to prolonged periods of neutropenia and heightened mucosal vulnerability caused by cytotoxic drugs [6]. In our case, the patient underwent chemo-radiation therapy twice for his gastric adenocarcinoma and urothelial carcinoma of the bladder. These treatment regimens significantly compromised the integrity of the oesophageal mucosa, rendering it highly susceptible to fungal infections. On the other hand, immunosenescence, the age-related decline in immune function, could have also played a role in the susceptibility to *Aspergillus* oesophagitis in our case. The probable route of infection in our situation can be through the inhalation or ingestion of *Aspergillus* spores that then reached the oesophagus and caused oesophagitis, which is a common route of infection in individuals with compromised immune systems. In addition, since *Aspergillus* species have a predilection for causing invasive infections in the respiratory tract, including the lungs and sinuses, and the patient had a history of squamous cell carcinoma of the lung and lung surgery, this may have provided an opportunity for the *Aspergillus* infection to spread from the respiratory system to the oesophagus. The occurrence of oesophageal aspergillosis in our patient, two decades after lung surgery for squamous cell carcinoma, is noteworthy. The extended interval between the initial lung malignancy and the development of oesophageal aspergillosis is consistent with the chronic nature of pulmonary aspergillosis, as reported by Marshall *et al*. in 2010 [10], and raises the hypothesis of a 20 year latency in our case. This case thus underscores the importance of considering *Aspergillus* as a potential pathogen in patients with a history of pulmonary disease, even in the context of delayed onset disease.

Within the existing literature on oesophageal aspergillosis, a 35-year-old man diagnosed with acute myeloblastic leukaemia (AML) M4 developed oesophageal aspergillosis following a bone marrow transplant in 1997. Gastroscopy revealed the presence of dichotomously branching septate hyphae, consistent with aspergillosis, and the patient responded well to treatment [8]. Similarly, in 2004, two cases of oesophageal aspergillosis were reported in patients with AML who presented with dysphagia and anorexia, and the diagnosis was confirmed through cytologic brushings of the oesophagus [6]. In 2005, a localized gastrointestinal invasive aspergillosis (oesophageal aspergillosis) was observed as an adverse outcome of AML induction therapy, and treatment with voriconazole appeared successful in preventing further infection progression despite the continuation of myelosuppressive chemotherapy [11]. In 2006, a 15-year-old boy with AML developed a fungal lung infection after achieving remission, which subsequently resolved with treatment. However, upon relapse, he experienced refractory epigastric pain accompanied by nausea, vomiting, and odynophagia. Upper gastrointestinal endoscopy revealed ulcerated lesions with *Aspergillus* infection [12]. Another case in 2008 involved an 18-year-old boy with acute lymphoblastic leukaemia (ALL) who exhibited symptoms of oesophageal involvement and tested positive for oesophageal aspergillosis, despite no radiologic evidence of pulmonary infection [7]. The most recent report of *Aspergillus* oesophagitis, to the best of our knowledge, dates back to 2014 and pertains to a 46-year-old female with AML who developed dysphagia [13]. On another hand, instances of invasive aspergillosis in individuals with solid tumours are infrequent, and they primarily affect the pulmonary region [14]. For instance, only a small number of extrapulmonary occurrences in patients with solid tumours were reported, and they were involving the brain [15].

The poor outcome in our case, compared to other patients in the literature with favourable outcomes using voriconazole, can be attributed to diagnostic delays and host factors such as the presence of multiple malignancies. Delayed diagnosis may have led to a delayed initiation of appropriate treatment, while the patient’s multiple malignancies and associated treatments may have severely compromised the immune system, impacting treatment response.

This case highlights the unusual presentation of oesophageal aspergillosis in a patient with multiple solid tumours, expanding our understanding of fungal infections in immunocompromised individuals beyond the typical haematological malignancy population. While the literature predominantly describes oesophageal aspergillosis in leukaemia patients, our case underscores the importance of considering this diagnosis in patients with solid tumours, particularly those with risk factors such as advanced disease, immunosuppression, and prior radiation therapy. Several factors also contributed to the severity of this case, including the patient’s complex medical history, delayed diagnosis, and the aggressive nature of the infection. This case emphasizes the need for heightened clinical suspicion of oesophageal aspergillosis in patients with similar risk profiles.

However, the current report is based on a retrospective analysis of only one patient and cannot draw definitive conclusions about the incidence, risk factors, and optimal management of oesophageal aspergillosis in this patient population. Moreover, while molecular techniques are undeniably superior for precise species identification and drug susceptibility testing, the current study relied on conventional diagnostic methods due to the limitations of the available clinical material. As such, the specific *Aspergillus* species involved in this case remains undetermined. This represents a limitation of the case, as the identification of cryptic species and determination of drug susceptibility profiles could have provided additional valuable insights into the pathogenesis and management of this case. Despite these limitations, our case offers valuable insights into the challenges of diagnosing and managing oesophageal aspergillosis in patients with multiple solid tumours. Indeed, early recognition of symptoms, such as dysphagia, odynophagia, and chest pain, is crucial for timely diagnosis and initiation of appropriate antifungal therapy. Future prospective research with large sample sizes should address these limitations by enhancing our understanding of this rare condition and exploring the potential benefits of prophylactic antifungal strategies in high-risk patients.

## Conclusion

Oesophageal aspergillosis, although rare, should be considered in immunocompromised cancer patients, who present with refractory symptoms such as epigastric pain, dysphagia, and gastrointestinal distress; where prompt recognition, accurate diagnosis through techniques like gastrointestinal endoscopy, and timely initiation of appropriate antifungal treatment are crucial for improved outcomes.

## Abbreviations

ALL: acute lymphoblastic leukaemia
AML: acute myeloblastic leukaemia
CRP: c-reactive protein
GEJ: gastro-oesophageal junction
IA: invasive aspergillosis
IV: intravenous
PAS: periodic acid-Schiff
PO: per os

## References

[1] Latgé J-P, Chamilos G (2019). *Aspergillus fumigatus* and Aspergillosis in 2019. Clin Microbiol Rev.

[2] Darling BA, Milder EA (2018). Invasive aspergillosis. Pediatr Rev.

[3] Eggimann P, Chevrolet J-C, Starobinski M, Majno P, Totsch M (2006). Primary invasive aspergillosis of the digestive tract: report of two cases and review of the literature. Infection.

[4] Gregg KS, Kauffman CA (2015). Invasive Aspergillosis: epidemiology, clinical aspects, and treatment. Semin Respir Crit Care Med.

[5] Yoo JH, Shin WS, Kim YR, Kang MW, Kim DW (1995). *Esophageal aspergillosis* in a patient with acute leukemia. Leukemia.

[6] Bergman S, Geisinger KR (2004). *Esophageal aspergillosis* in cytologic brushings: report of two cases associated with acute myelogenous leukemia. Diagn Cytopathol.

[7] Akyol Erikci A, Ozyurt M, Terekeci H, Ozturk A, Karabudak O (2009). *Oesophageal aspergillosis* in a case of acute lymphoblastic leukaemia successfully treated with caspofungin alone due to liposomal amphotericin B induced severe hepatotoxicity. Mycoses.

[8] Choi JH, Yoo JH, Chung IJ, Kim DW, Han CW (1997). *Esophageal aspergillosis* after bone marrow transplant. Bone Marrow Transplant.

[9] Sridhar H, Jayshree RS, Bapsy PP, Appaji L, Navin Kumar M (2002). Invasive aspergillosis in cancer. Mycoses.

[10] Marshall H, Jones S, Williams A (2010). Chronic pulmonary aspergillosis - longterm follow-up over 20 years, a case report. J Radiol Case Rep.

[11] Chionh F, Herbert KE, Seymour JF, Prince HM, Wolf M (2005). Ante-mortem diagnosis of localized invasive esophageal aspergillosis in a patient with acute myeloid leukemia. Leuk Lymphoma.

[12] Alioglu B, Avci Z, Canan O, Ozcay F, Demirhan B (2007). Invasive esophageal aspergillosis associated with acute myelogenous leukemia: successful therapy with combination caspofungin and liposomal amphotericin B. Pediatr Hematol Oncol.

[13] Besa S, Kattan E, Cid X, Claro JC (2014). *Aspergilosis esofágica* en una paciente con leucemia mieloide aguda y neutropenia febril. Rev chil infectol.

[14] Dandachi D, Wilson Dib R, Fernández-Cruz A, Jiang Y, Chaftari A-M (2018). Invasive pulmonary aspergillosis in patients with solid tumours: risk factors and predictors of clinical outcomes. Ann Med.

[15] Ohmagari N, Raad II, Hachem R, Kontoyiannis DP (2004). Invasive aspergillosis in patients with solid tumors. Cancer.

